# RAGE contributes to allergen driven severe neutrophilic airway inflammation *via* NLRP3 inflammasome activation in mice

**DOI:** 10.3389/fimmu.2023.1039997

**Published:** 2023-01-26

**Authors:** Katherine N. Killian, Jessica L. Kosanovich, Madeline A. Lipp, Kerry M. Empey, Tim D. Oury, Timothy N. Perkins

**Affiliations:** ^1^ Department of Pathology, University of Pittsburgh, School of Medicine, Pittsburgh, PA, United States; ^2^ Department of Pharmaceutical Sciences, University of Pittsburgh, School of Pharmacy, Pittsburgh, PA, United States; ^3^ Department of Pharmacy and Therapeutics, University of Pittsburgh, School of Pharmacy, Pittsburgh, PA, United States; ^4^ Center for Clinical Pharmaceutical Sciences, University of Pittsburgh, School of Pharmacy, Pittsburgh, PA, United States; ^5^ Department of Immunology, University of Pittsburgh, School of Medicine, Pittsburgh, PA, United States

**Keywords:** RAGE (receptor for advanced glycation end products), NLRP3, asthma, allergen, neutrophil, alternaria

## Abstract

**Background:**

Asthma is a major public healthcare burden, affecting over 300 million people worldwide. While there has been great progress in the treatment of asthma, subsets of patients who present with airway neutrophilia, often have more severe disease, and tend to be resistant to conventional corticosteroid treatments. The receptor for advanced glycation endproducts (RAGE) plays a central role in the pathogenesis of eosinophilic asthma, however, it’s role in neutrophilic asthma remains largely uninvestigated.

**Methods:**

A mouse model of severe steroid resistant neutrophilic airway disease (SSRNAD) using the common fungal allergen *Alternaria alternata* (AA) was employed to evaluate the effects of genetic ablation of RAGE and pharmacological inhibition of the NLRP3 inflammasome on neutrophilic airway inflammation.

**Results:**

AA exposure induced robust neutrophil-dominant airway inflammation and increased BALF levels of Th1/Th17 cytokines in wild-type mice, which was significantly reduced in RAGE^-/-^ mice. Serum levels of IgE and IgG1 were increased similarly in both wild-type and RAGE^-/-^ mice. Pharmacological inhibition of NLRP3 blocked the effects of AA exposure and NLRP3 inflammasome activation was RAGE-dependent. Neutrophil extracellular traps were elevated in the BALF of wild-type but not RAGE^-/-^ mice and an atypical population of SiglecF+ neutrophils were identified in the BALF. Lastly, time-course studies found that RAGE expression promoted sustained neutrophil accumulation in the BALF of mice in response to AA.

## Introduction

Asthma remains a major public health burden, affecting nearly 350 million people worldwide and its prevalence is steadily increasing (1). Asthma is a generalized term used to describe a range of disease phenotypes of variable severity, which share a common characteristic of reversible airflow obstruction (2, 3). Asthma is caused by both genetic predisposition and exposure to environmental triggers (e.g. allergens, air pollution and cigarette smoke), which can be associated with different phenotypes. A growing number of populations with more severe disease are resistant to conventional therapies and need novel preventative and therapeutic intervention strategies. Approximately half of moderate to severe cases of asthma present with a “type 2-high” (T2-hi) phenotypic signature, with elevated type 2 cytokines and often increased IgE and eosinophilia (4–7). Great progress has been made in the treatment of T2-hi asthma, with the success of various biologics targeting type 2 inflammation (8). Conversely, “type 2-low” (T2-lo) asthma remains difficult to treat, as many subjects have more severe disease, frequent exacerbations and resistance to corticosteroids (9). The mechanisms of T2-lo asthma remain poorly understood, however, it often exhibits increased airway neutrophils, Th1/Th17 cytokines and is associated with frequent lung infections, air pollution and obesity (10–12).

Innate immune sensors play a critical role in protecting the lungs from exogenous pathogens and pollutants (13, 14). Recent clinical and experimental studies have implicated an important role for the nucleotide-binding oligomerization domain (NOD)-like receptor family pyrin domain containing 3 (NLRP3) inflammasome in neutrophilic asthma (15–18). The NLRP3 inflammasome is a multi-protein immune sensor complex which consists of three subunits: NLRP3, the adaptor subunit (ASC) and pro-caspase-1 subunit. Activation, which is a two-step process, promotes the processing of inflammatory cytokines IL-1β and IL-18 into active forms. In step one, the “priming” step, pathogen-associated molecular patterns (PAMPs) or pro-inflammatory cytokines (e.g. TNFα) induce expression of the subunits pro-IL-1β/pro-IL-18. Subsequently, PAMPs or damage-associated molecular patterns (DAMPs) signal through pattern-recognition receptors (PRRs) to induce assembly and activation of the inflammasome, leading to cleavage of pro-IL-1β and pro-IL-18, which promotes Th1 and Th17 immune responses (19–23).

The receptor for advanced glycation endproducts (RAGE) is a member of the immunoglobulin superfamily of proteins, which binds a multitude of ligands, primarily DAMPs such as HMGB1 and is most abundantly expressed in the lungs (24–27). RAGE signaling drives inflammation in various chronic conditions including diabetes and chronic obstructive pulmonary disease (22, 23). Our laboratory and others have previously demonstrated that RAGE is a critical mediator of Th2 inflammation in various experimental models of asthma (28–33). Moreover, clinical studies have shown that various RAGE ligands are elevated in asthmatics, while levels of soluble (s)RAGE (a decoy receptor) are decreased [reviewed in (34)]. Recent human genomic studies suggest that reduced sRAGE levels likely play a causal role in asthma (35–37). Notably, studies have shown that sRAGE levels are significantly reduced in subjects with neutrophilic asthma and COPD compared to those without neutrophilia and that sRAGE levels have a positive correlation with lung function and a negative correlation with neutrophil numbers (38). This suggests that increased RAGE signaling may promote neutrophilic inflammation in asthma.

The present study examines the role of RAGE in an experimental model of allergen-driven severe steroid-resistant neutrophilic airway disease (SSRNAD). This study demonstrates that RAGE expression promotes neutrophil-dominant airway inflammation in response to *Alternaria alternata* (AA) exposure. In addition, AA-induced increases in Th1 and Th17 cytokines and chemokines are markedly reduced in RAGE-deficient mice. Moreover, this study demonstrates that RAGE expression is required for NLRP3 inflammasome activation and IL-1β release, while pharmacological inhibition of NLRP3 significantly reduces neutrophilic inflammation and Th1/Th17 cytokines in the airways. Furthermore, the present study identified the presence of an understudied population of atypical neutrophils and that neutrophil extracellular traps (NETs) accumulate in the airways in this experimental model of SSRNAD. This study demonstrates that RAGE is a critical mediator of NLRP3-dependent, allergen-driven, severe neutrophilic airway inflammation.

## Materials and methods

### Animals

Wild-type (WT) C57BL/6N mice were purchased from Taconic (Hudson, NY). RAGE-knockout (RAGE^-/-^) mice were a king gift from Dr. A. Bierhaus (University of Heidelburg, Heidelburg, Germany) and a breeding colony was established (39). Both male and female mice were used at eight to ten weeks of age in all animal experiments. All animal studies were carried out under the National Research Council’s guidelines in the ‘‘Guide for the care and use of laboratory animals,’’ with oversight by the University of Pittsburgh’s Institutional Animal Care and Use Committee.

### Models of SSRNAD

An experimental model of SSRNAD was adapted from previously established models (40, 41). *Alternaria alternata* extract (XPM1D3A25) was purchased from Greer labs (Lenoir, NC) and reconstituted in sterile saline based on protein content. On day 0, mice were sensitized by subcutaneous (s.c) injection on the dorsal surface with an emulsion of 50µl Complete Freund's Adjuvant (CFA) (Sigma) in 50µl of saline containing 100µg AA or saline alone (100µl total volume). Mice were then challenged daily by intranasal (i.n.) administration of 25µg AA in 40µl saline or saline alone on days 14-17 and then euthanized 24h later. In additional experiments, mice receive an i.p. injection of 1% DMSO in saline (vehicle control) or 2mg/kg dexamethasone immediately prior to i.n. challenge on day 14 and 16 as previously described (41). In other experiments, mice were i.n. challenged on days 14-17 with 25µg AA with 10% DMSO in saline (vehicle) or the NLRP3 inhibitor, MCC950 (10mg/kg). In time-course experiments, mice are sensitized to AA/CFA (100µg) on day 0 and 14 days later challenged once i.n with 25µg AA or saline and mice were euthanized at, 1h, 3h, 6h, 12h or 24h post-challenge.

### Bronchoalveolar lavage fluid

Mouse lungs were lavaged with 0.8ml saline solution and cytospin preps were prepared and total and differential cell counts were performed as previously described (28). Cells were pelleted and supernatant separated, and specimens were stored at -80°C until further analysis. Cytokines were measured in BALF supernatant by ELISA following manufacturer’s protocol. ELISA kits for mouse CXCL1, G-CSF, IFNγ, IL-1β, IL-4, IL-5, IL-6, neutrophil elastase and S100A8/S100A9 were purchased from R&D (Duoset), IL-17A from Biolegend (ELISAMAX) and IL-13 from Invitrogen.

### Measurement of serum immunoglobulins

Non-specific mouse IgE and IgG_1_ were measured by ELISA (Invitrogen) according to manufacturer’s protocol. For IgE serum was diluted 1:20 in assay buffer and for IgG_1_, 1:50 in assay buffer. For AA-specific IgE and IgG_1_, microplates were coated with 25µg/ml AA extract in PBS at room temperature overnight. Plates were washed three times with 0.1% PBS-Tween (PBS-T), and then incubated with serum (1:20 for IgE and 1:50 for IgG_1_) overnight at 4°C. Samples were discarded and plates washed three times with PBS-T. For AA-specific IgE, the IgE ELISA kit manufacturer’s protocol was followed beginning with the detection antibody step. For AA-specific IgG_1_, plates are incubated with donkey anti-mouse IgG_1_-HRP (Jackson Immunoresearch) diluted 1:1000 in 1% BSA-PBS for 1h at RT in the dark. Plates are washed and then incubated with SigmaFast OPD substrate for 30min at RT in the dark. Stop solution (1M HCl) was added to plates and absorbance was read at 450nm.

### Western blotting

Whole lungs were homogenized in 4%CHAPS lysis buffer and mixed 1:1 in CHAPS-Urea buffer (9M urea, 4%CHAPS 10mM Tris) as previously described (30). Pelleted BAL cells were lysed in CHAPS urea buffer. Homogenates, lysates and BALF were mixed 3:1 in 4X laemmli buffer (Bio-rad) supplemented with 50mM DTT and boiled for five minutes. 20-60µg of protein or 5µl BALF was loaded on 10% SDS-PAGE gels (TGX Fastcast, Bio-rad) and run at 100V for ten minutes followed by 150V for an additional 45 minutes. Gels were then transferred onto PVDF membranes (Millipore) at 100V for one hour on ice. Blots were then blocked in 2.5% BSA-PBS for one hour at room temperature. Blots were incubated in primary antibody diluted in 2.5% BSA-PBS overnight at 4°C. Antibodies used include anti-NLRP3, anti-pSTAT3, anti-STAT3, anti-β-Actin (Cell signaling technologies), anti-Caspase-1 (Santa Cruz Biotechnology), anti-HMGB1 and anti-citrullinated-Histone H3 (Abcam) and anti-RAGE antibody (in house). Blots were then washed in PBS-T (0.1% Tween-20) 3X for 10 minutes. Blots were then incubated with HRP-conjugated secondary antibodies diluted in 2.5% non-fat milk-PBS for 1h at room temperature. Blots were again washed in PBS-T 3X for 10 minutes. Blots were incubated with Pierce ECL substrate (Thermo Scientific) and were imaged on a LI-COR Odyssey Fc by using LI-COR Image Studio software (LI-COR Biosciences).

### Flow cytometric analysis

Cells collected from the BAL were surface stained with CD16/32 (Fc block; 2.4G2), LIVE/DEAD™ Fixable Blue Dead Cell Stain kit (ThermoFisher Scientific, Waltham, MA), CD11b PerCP/Cy5.5 (M1/70), Ly6G BV785 (1A8), SiglecF APC/Cy7 (E50-2440), F4/80 BV421 (T45-2342; BD Biosciences), CD200R PE (OX-110; BioLegend), CD11c PE/Cy7 (N418; BioLegend) and CD206 APC (C068C2; BioLegend) prior to fixation with BD Cytofix™ (BD Biosciences, San Jose, CA). Samples were collected on a Cytek Aurora managed by the United Flow Core of the University of Pittsburgh and data was analyzed using FlowJo V10 software (FLOWJO, LLC, OR).

### Statistical analysis

Statistical analyses were performed with GraphPad Prism 9 software. Data are expressed as mean ± SEM. Statistical significance was determined by using One-way ANOVA with a Tukey post-test for multiple comparisons or an unpaired Student’s t-test, where appropriate. A P-value of less than 0.05 was considered statistically significant.

## Results

### RAGE promotes AA-induced steroid-resistant neutrophil-dominant airway inflammation in mice

Previous studies demonstrated that i.n. challenge with house dust mite (HDM) after prior sensitization in the presence of complete Freund’s adjuvant (CFA) leads to dexamethasone-resistant neutrophil-dominant inflammation (40, 41). *Alternaria alternata* (AA) is a common fungal allergen associated with severe asthma and exacerbations (42–44). In the present study, mice were sensitized to AA extract in the presence of CFA 14 days prior to four consecutive daily i.n. challenges with AA or saline alone. To confirm that this AA/CFA model is also steroid-resistant, mice were treated by i.p. injection with either vehicle control or dexamethasone (DEX) on days 14 and 16. (**Figure S1A**) Challenge with AA induced robust neutrophil-dominant inflammation in the BALF (**Figure S1B**) and increased levels of Th1/17 cytokines and chemokines (**Figures S1C–G**), IL-17A, IFNγ, IL-6, G-CSF and CXCL1. Treatment with DEX did not reduce neutrophil accumulation or levels of Th1/17 inflammatory mediators (IL-17A, IFNγ, IL-6, G-CSF and CXCL1) in the BALF of WT mice. (**Figures S1B–G**).

**Figure 1:**
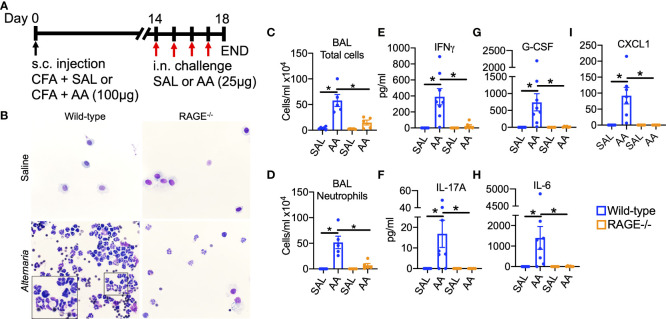
RAGE promotes AA-induced neutrophil-dominant airway inflammation in mice. Experimental design: WT and RAGE^-/-^ mice were sensitized to saline or **(A)** A extract (100µg) in the presence of CFA on day zero. Mice were then intranasally challenged with saline or AA (25µg) daily on days 14-17 and then euthanized 24h after the final challenge. **(B)** Representative photomicrographs of cytospin preps. **(C)** Total cells/ml and **(D)** neutrophils/ml in BALF quantified by total and differential cell counts. **(E-I)** BALF levels of **(D)** IL-17A, **(E)** IFNγ, **(F)** IL-6, **(G)** G-CSF and **(H)** CXCL1. Data are represented as the mean ± SEM, N=5-8 per group. Data are from a single experiment and are representative of 2-3 independent experiments. *P<0.05 for indicated comparison.

To determine the effects of RAGE expression on airway neutrophilia and Th1/17 cytokine/chemokine elaboration, WT and RAGE^-/-^ mice were subjected to the AA/CFA model of SSRNAD (**Figure 1**). WT mice challenged with AA exhibited robust neutrophil-dominant inflammation the BALF, which was significantly abrogated in RAGE^-/-^ mice. (**Figures 1A–D**). Moreover, elevated levels of Th1/17 cytokine/chemokine levels were nearly completely abolished in RAGE^-/-^ mice. (**Figures 1E–I**) BAL cell differential counts revealed that AA-induced inflammation was approximately 80% and 50% neutrophils in WT and RAGE^-/-^ mice, respectively, with corresponding reductions in the percent of macrophage/monocytes, which are the primary cell present in naïve or saline challenged mice. (**Figures S2A, B**) Eosinophils and lymphocytes both only accounted for less than 5 percent of cells in the BALF. (**Figures S2C, D**) There were no significant changes in the number of macrophage/monocytes or lymphocytes. (**Figures S2E, F**) Exposure to AA alone (without CFA), typically induces robust eosinophilic inflammation and increased levels of type 2 cytokines IL-5 and IL-13 (29). In the current model, AA challenge induced a statistically significant increase in BAL eosinophils in WT but not RAGE^-/-^ mice (**Figure S2G**) although, these levels were far less than those typically induced by challenge with AA alone (29). Interestingly, AA challenge caused a trend towards elevated levels of IL-5 and IL-13 in the BAL of both WT and RAGE^-/-^ mice, however, this failed to reach statistical significance (**Figures S2H, I**). These data demonstrate that RAGE expression promotes AA-induced steroid-resistant and neutrophil-dominant airway inflammation in mice.

**Figure 2:**
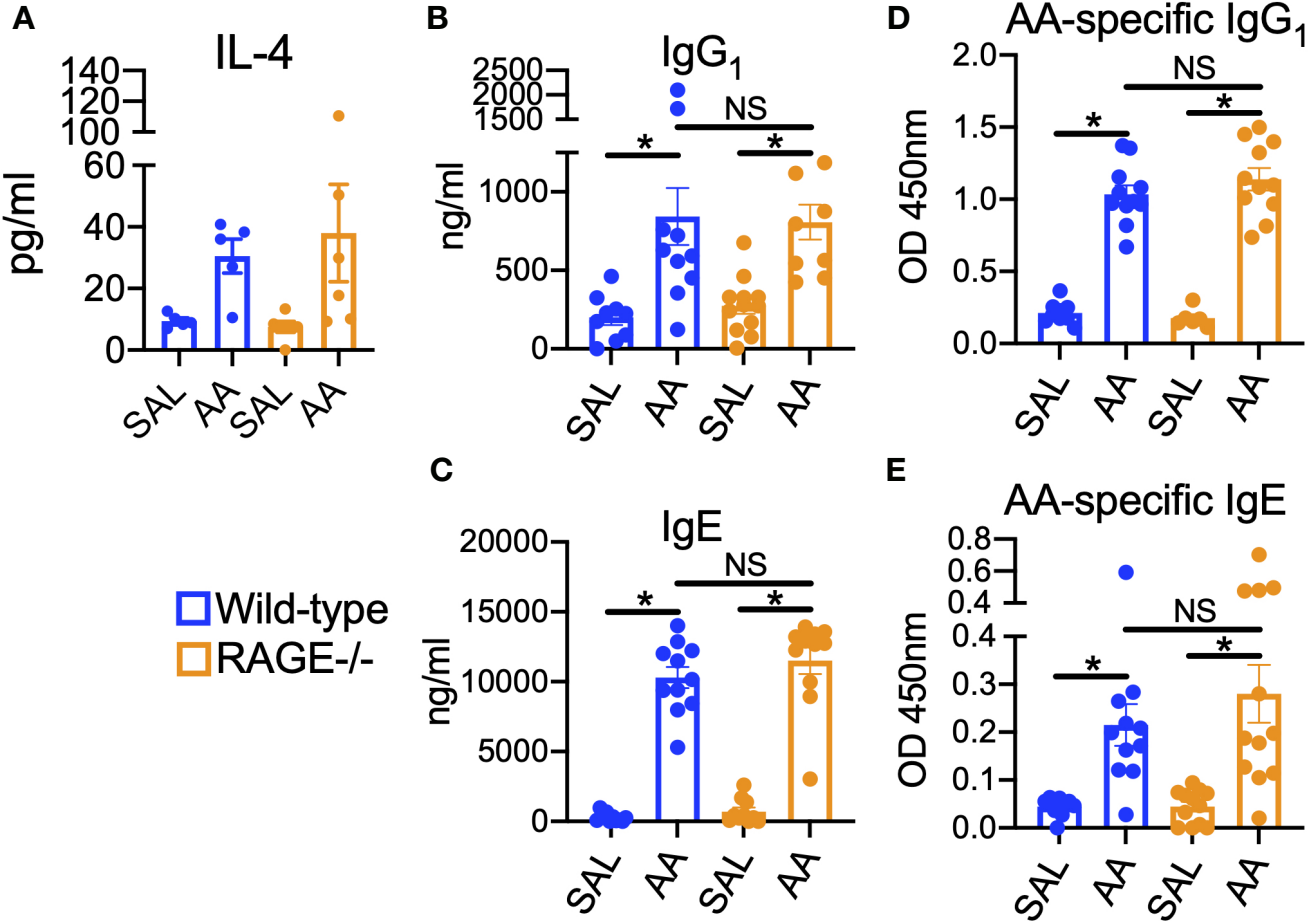
Humoral immune responses to AA remain intact in RAGE^-/-^ mice. WT and RAGE^-/-^ mice were subjected to the AA/CFA model. **(A)** BALF levels of IL-4. Serum levels of non-specific **(B)** IgG^1^ and **(C)** IgE. Serum levels of AA-specific **(D)** IgG1 and **(E)** IgE. Data are represented as the mean ± SEM. For IL-4, N=5-6 per group and data are from a single experiment. For Immunoglobulins, N=9-6 per group and data are pooled from two independent experiments. *P < 0.05 for indicated comparison.

Interestingly, the humoral immune response to AA remained intact in RAGE^-/-^ mice (**Figure 2**). Levels of IL-4 showed a trend in elevation in the BALF of both WT and RAGE^-/-^ mice (**Figure 2A**), which regulates Immunoglobulin (Ig) class switching. Similarly, both WT and RAGE^-/-^ mice challenged with AA produced similar circulating levels of non-specific (**Figures 2B, C**) and AA-specific (**Figures 2D, E**) IgE and IgG_1_. These data are consistent with previous findings showing that humoral immune responses in a model of chronic HDM exposure were independent of RAGE (28).

### RAGE promotes allergen-induced NLRP3 inflammasome activation

**Figure 3:**
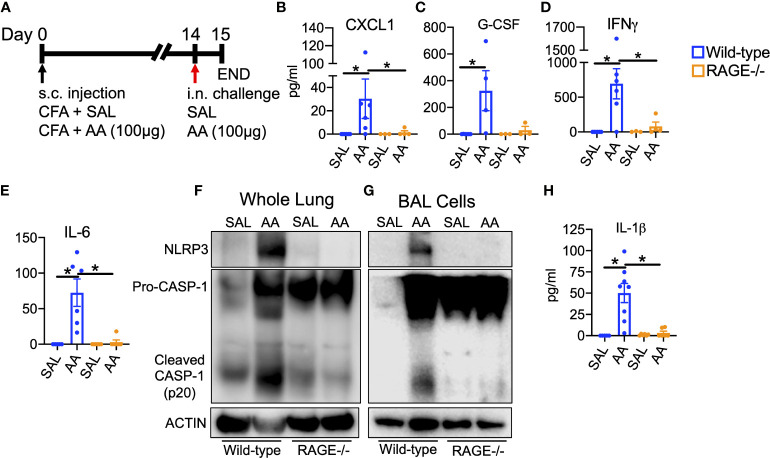
RAGE promotes allergen-driven NLRP3 inflammasome activation **(A)** WT and RAGE^-/-^ mice were sensitized to saline or AA extract (100µg) in the presence of CFA on day zero and then intranasally challenged once with saline or AA (100µg) and then euthanized 24h after challenge. BALF cytokine levels of CXCL1 **(B)**, G-CSF **(C)**, IFNγ **(D)** and IL-6 **(E)** in WT and RAGE^-/-^ mice after single high dose challenge. Western blots of whole lung homogenate **(F)** or BAL cell lysates **(G)** for NLRP3, Caspase-1 and Actin. Each lane represents 3-4 pooled biological replicates. **(H)** BALF levels of IL-1β from WT and RAGE^-/-^ mice after multiple challenges as depicted in **Figure 1A**. N=3-8 biological replicates per group for ELISA data. *P<0.05.

**Figure 4:**
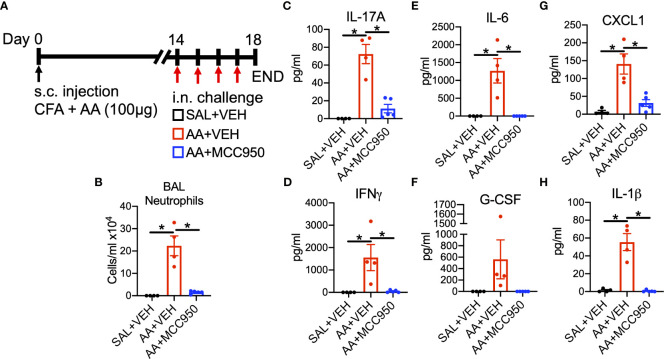
Pharmacological antagonism of NLRP3 inhibits allergen-driven neutrophil-dominant airway inflammation **(A)** WT mice were sensitized to AA extract (100µg) or saline (control) in the presence of CFA on day zero. Mice are then co-administered saline + vehicle (10%DMSO), AA (25µg) + vehicle or AA + MCC950 (10mg/kg) daily on days 14-17 and then euthanized 24h after the final challenge. **(B)** Total neutrophils/ml in BALF quantified by total and differential cell counts. BALF levels of **(C)** IL-17A, **(D)** IFNγ, **(E)** IL-6, **(F)** G-CSF, **(G)** CXCL1 and **(H)** IL-1β. Data are represented as the mean ± SEM, N=4-5 per group. Data are from a single experiment and are representative of 2-3 independent experiments. *P<0.05 for indicated comparison.

Several early secreted factors promote polarization and activation of innate and adaptive immune cells. IL-23, IL-6, TGFβ and IL-1β are known to promote polarization of type 17 immune responses (45, 46). Therefore, activation of upstream and downstream signaling pathways associated with these secreted factors were examined in WT and RAGE^-/-^ mice after a single high dose challenge of AA (**Figure 3A**). Although IL-17A was not detectable in BALF after a single challenge (data not illustrated), levels of IL-17A-inducible cytokines, CXCL1 and G-CSF, as well as the Th1 cytokine IFNγ were elevated in the BALF of WT, but not RAGE^-/-^ mice (**Figures 3B–D**). IL-23 levels were not detected in the BALF after a single or multiple AA challenges (data not illustrated); however, IL-6 was elevated in the BALF of AA challenged WT, but not RAGE^-/-^ mice (**Figure 3E**). IL-6 and IL-23 promote type 17 inflammation *via* STAT3-signaling (47, 48), which was also activated as indicated by increased phospho(p)STAT3 levels in whole lung homogenate (WLH) and BAL cell lysates of WT, but not RAGE^-/-^ mice (**Figure S3**). In addition, levels of NLRP3 and cleaved Caspase-1 (p20) protein were elevated in WLH and BAL cell lysates of WT, but not RAGE^-/-^ mice after AA challenge (**Figures 3F, G**). Interestingly, baseline levels of pro-caspase-1 were elevated in saline challenged RAGE^-/-^ mice compared to WT mice (**Figures 3F, G** and **Figure S4**), however there was no increase in cleaved Caspase-1 (p20) in RAGE^-/-^ mice. Although levels of IL-1β were not detected after a single challenge with AA, after 4 daily challenges, levels of IL-1β were significantly elevated in the BALF of WT, but not RAGE^-/-^ mice (**Figure 3H**). DEX treatment also did not reduce levels of IL-1β in the BALF of WT mice (**Figure S1H**). Importantly, treatment with a specific pharmacological inhibitor of NLRP3 (MCC950), significantly abrogated airway neutrophilia and inflammatory cytokine and chemokine secretion in the BALF of WT mice challenged with AA (**Figures 4A–H**). Treatment with MCC950 had no effect on circulating levels of non-specific IgE and IgG_1_. (**Figure S5**). These data suggest that RAGE promotes AA-induced neutrophilic inflammation by promoting STAT3-signaling and NLRP3 inflammasome activation.

### NETs accumulate in the lungs of mice in the AA/CFA model of SSRNAD

**Figure 5:**
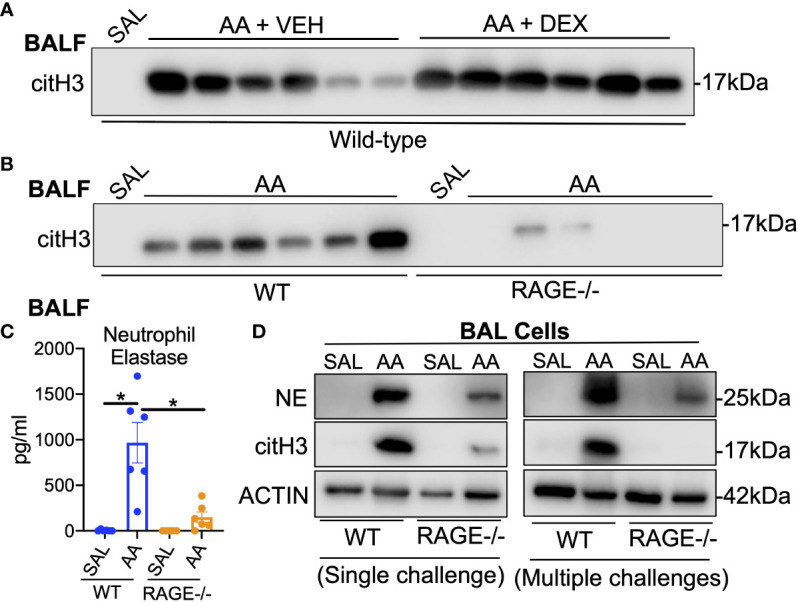
NETs accumulate in the lungs of mice in the AA/CFA model of SSRNAD Western blot of citrullinated histone H3 (citH3) cell free BALF specimens (5µl per lane) from **(A)** WT mice challenged with saline treated with vehicle (N=6 pooled biological replicates), AA + vehicle or AA + Dexamethasone (N=6) as described in **Figure S1**, **(B)** from WT and RAGE^-/-^ mice challenged with saline (N=5 pooled biological replicates) or AA (N=6) as described in **Figure 1**. **(C)** BALF levels of Neutrophil elastase (NE) as measured by ELISA (N=5-6, *P<0.05). Western blot of BAL cells from **(D)** single AA challenge (left) or 4 daily AA challenges (right). Blots were probed for NE, citH3 or Actin. Each lane represents 3-6 pooled biological replicates. Data are from a single experiments and are representative of 2 independent experiments.

Due to the massive influx of neutrophils into the airways, the presence of NETs was assessed. Indeed, levels of citrullinated histone H3 (citH3), a marker of NETs (49), were elevated in the BALF of WT mice exposed to AA, which was not reduced by DEX treatment (**Figure 5**). In addition, levels of citH3 and neutrophil elastase (NE) were markedly reduced in the BALF of RAGE^-/-^ mice (**Figures 5B, C**). Moreover, levels of NE and citH3 were elevated in BAL cell lysates of WT mice after single or multiple AA challenges, which was reduced in RAGE^-/-^ mice (**Figure 5D**). Several antimicrobial molecules are also released with NETs during NETosis, including the heterodimer S100A8/S100A9 (S100A8/A9) (50). Interestingly, the levels of the S100A8/A9 were elevated similarly in the BALF of both WT and RAGE^-/-^ mice in the AA/CFA model (**Figure S6A**). However, in a model of AA exposure alone (29), S100A8/A9 levels were elevated in the BALF of WT but not RAGE^-/-^ mice (**Figure S6B**).

### Atypical neutrophils are recruited to the airways of mice in response to AA

**Figure 6:**
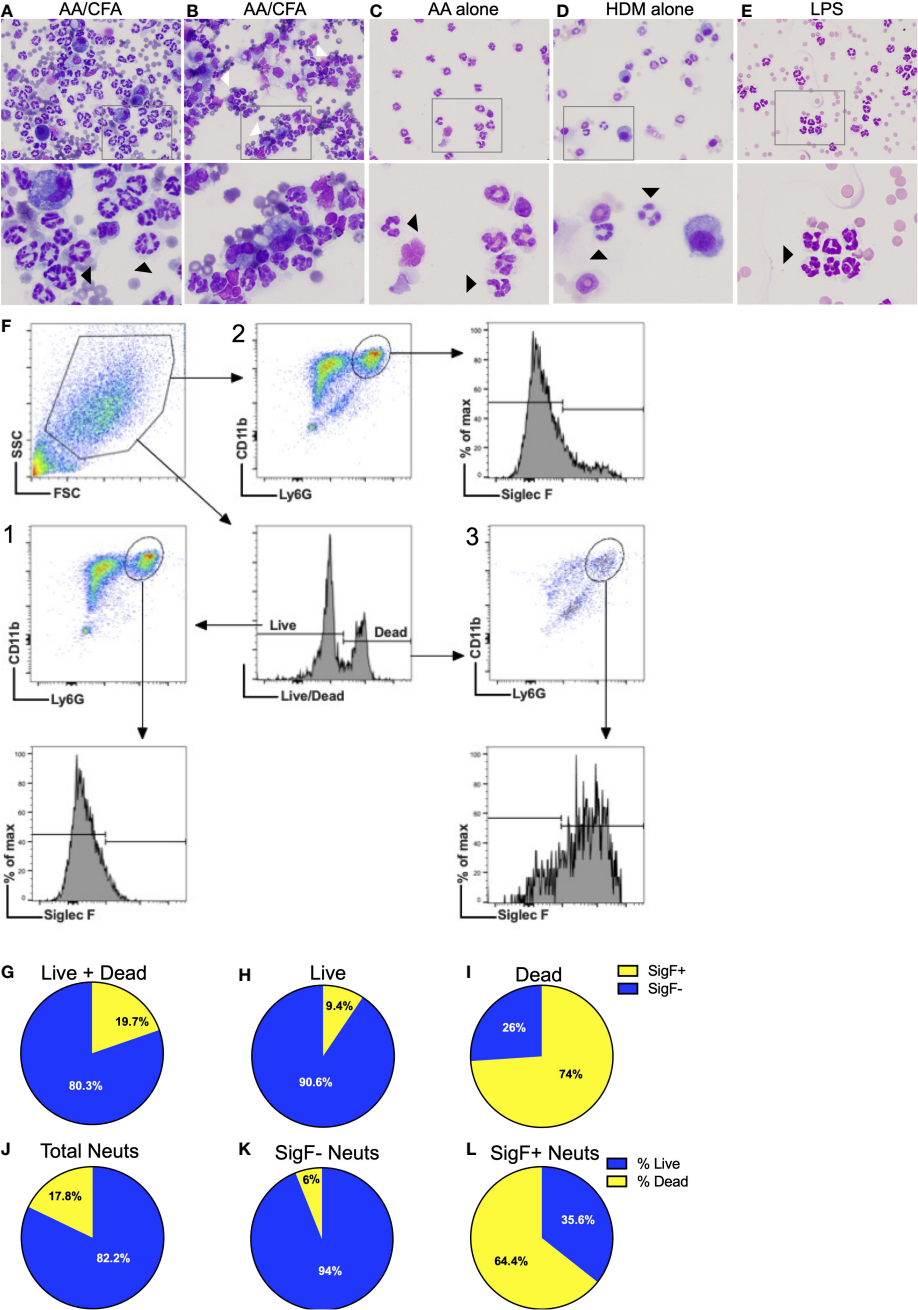
Atypical Neutrophils recruited to the airways exhibit abnormal morphology **(A, B)** representative photomicrographs of BALF cytospin preps from WT mice exposed to AA/CFA, **(C)** AA alone, **(D)** HDM alone, or **(E)** LPS. Black arrows indicate neutrophils, white arrows indicate clumping cells. **(F)** Representative flow plots of neutrophil gating strategy. SSC^Hi^ cells were sequentially gated using Live/Dead (1, 3) or CD11b/Ly6G (2) plots. Resulting neutrophils, defined as CD11b+ Ly6G+ were then assessed for SiglecF expression using FMO controls. Ratio of SigF+ (yellow) and SigF- (blue) BALF neutrophils which were **(G)** Live+Dead, **(H)** Live only and **(I)** Dead only. Ratio of live neutrophils (blue) and dead neutrophils (yellow) for **(J)** total neutrophils, **(K)** SigF- neutrophils and **(L)** SigF+ neutrophils. N=4/group.

Further examination of BALF cytospin preparations revealed that neutrophils recruited to the airways in the AA/CFA model exhibited atypical morphology. Neutrophils had hyper-segmented nuclei, up to 12 lobes (**Figure 6A**) and clumping of cells (**Figure 6B**) as compared to neutrophils in the BAL of mice exposed to AA, HDM or LPS alone (**Figure 6C–E**). A recent report (51) found that diesel exhaust particle exposure induced recruitment of a unique population of SiglecF-expressing neutrophils, to airways of mice, which had atypical morphology. Interestingly, there was a population of SiglecF+ neutrophils in the BAL of AA/CFA challenged mice (**Figures 6F–L**). Notably, when gating for live cells only, SiglecF+ neutrophils accounted for 9.4% of total neutrophils (**Figures 6F1, G**), when gating for both live/dead cells, SiglecF+ neutrophils accounted for 19.7% of total neutrophils (**Figures 6F2, H**) and 74% of dead neutrophils (**Figure 6F3, I**). Moreover, with 17.8% of total neutrophils dead, only 9% of SiglecF- neutrophils were dead, as compared to 64.4% of SiglecF+ neutrophils. (**Figures 6L**) To account for potential background signal or non-specific binding of anti-SiglecF by dead cells, fluorescence minus one (FMO) controls for anti-SiglecF had no background signal (**Figure S7A**) and dead lymphocytes, which do no not express SiglecF did not non-specifically bind anti-SiglecF (**Figure S7B**). Additionally, neutrophils do not express F4/F80, whereas alveolar macrophages do (52) and while macrophages showed a positive shift in F4/F80 signal, dead neutrophils did not (**Figure S7C**). This suggests that SiglecF expression may mark late-stage neutrophils, which may be going through NETosis or another form of cell death.

### RAGE promotes persistent neutrophilic airway inflammation in response to allergen

**Figure 7:**
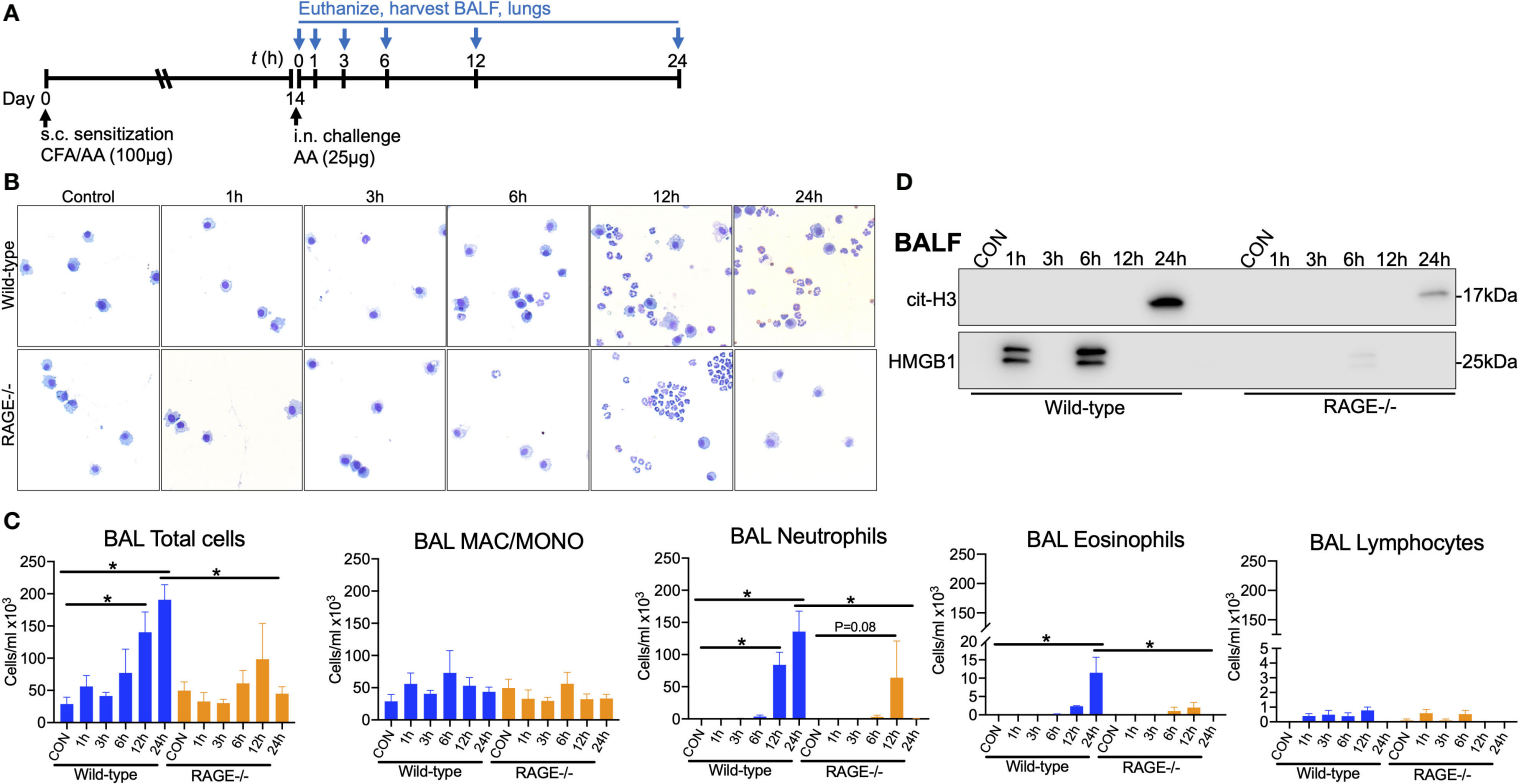
RAGE promotes early DAMP release and persistent neutrophilic airway inflammation in response to AA. **(A)** WT and RAGE^-/-^ mice were sensitized to saline or AA (100µg) in the presence of CFA on day 0. On day 14, mice were challenge once with saline or AA (25µg) and euthanized at 1, 3, 6, 12 and 24h post challenge. **(B)** Representative photomicrographs of cytospin preps. **(C)** BALF total cell counts/ml and differential counts for macrophage/monocytes, neutrophils, eosinophils and lymphocytes. **(D)** Western blots for citH3 and HMGB1 in the BALF (5µl pooled specimens). Data from a single experiment and are representative of 2 independent experiments. N=4 per group, *P<0.05.

Time-course experiments were performed to determine the kinetics of inflammatory cell infiltration into the airways of WT and RAGE^-/-^ mice. Two weeks after sensitization, mice were challenged once with 25µg AA and euthanized at 1, 3, 6, 12 and 24h post-challenge (**Figure 7A**) There were significant increases in total cells in the BALF of WT mice at 12 and 24h, which was primarily neutrophils, with some eosinophils at 24h (**Figures 7B, C**). At 12h RAGE^-/-^ mice had similar BALF neutrophils numbers the compared to WT mice, which were markedly diminished by 24h (**Figures 7B, C**). Moreover, there was accumulation of cit-H3 in the BALF of WT mice by 24h, which was markedly reduced in RAGE^-/-^ mice. In addition, there was a biphasic accumulation of HMGB1 in the BALF of WT mice at 1h and 6h post-challenge, which was almost completely abrogated in RAGE^-/-^ mice (**Figure 7D**). These data suggest that RAGE promotes sustained neutrophilic inflammation and early HMGB1 release in response to AA in mice.

## Discussion

It has been well demonstrated that RAGE is a critical mediator of Th2-high eosinophilic airway inflammation in various experimental mouse models of asthma (28–33). However, decreased levels of sRAGE and increased levels of RAGE ligands in neutrophilic asthmatics suggests that RAGE signaling may contribute to neutrophilia in severe asthma (34). Nonetheless, the mechanistic role of RAGE in experimental models of SSRNAD remains largely under-investigated. The present study demonstrates that RAGE is a critical mediator of allergen-driven neutrophil-dominant airway inflammation in a mouse model of SSRNAD.

In the present study, mice were sensitized to AA extract in the presence of CFA, which skewed the immune response to a mixed Th1/Th17-high neutrophil-dominant inflammatory response. AA challenge induced robust neutrophil accumulation and increased levels of Th1/Th17 cytokines and chemokines in the BALF, which was resistant to dexamethasone treatment. Intriguingly, these AA-induced effects were significantly abrogated in RAGE-null mice. These data indicate that RAGE is a critical mediator of allergen-driven neutrophilic inflammation and Th1/Th17 cytokine increases in an experimental model of SSRNAD. A similar study in rats sensitized to ovalbumin with CFA, demonstrated that administration of the RAGE antagonist FPS-ZM1, modestly reduced ovalbumin induced inflammation (53). However, that model exhibited only 20 percent neutrophils (compared to 80% here) with no evidence of steroid-insensitivity (53).

Challenge with AA after sensitization in the presence of CFA as opposed to challenge with AA alone resulted in suppression of the typical robust eosinophilic type 2 immune response. Although some eosinophils were present, the number of eosinophils in the BAL was far less than that typically elicited by AA challenge (29). Moreover, although there was a non-significant trend in elevation of IL-5 and IL-13 in the BALF of both WT and RAGE^-/-^ mice, there was no significant increase in eosinophils in RAGE^-/-^ mice. Even if these levels were biologically relevant, this finding would be consistent with the findings of a previous study in which administration of recombinant IL-5 and IL-13 to WT and RAGE^-/-^ mice resulted in increased airway eosinophils in WT but not RAGE^-/-^ mice (30).

Although the direct effects of IL-17A on airway neutrophilia were not examined in the present study, previous studies demonstrated that IL-17A neutralization significantly reduced airway neutrophilia in mouse models of HDM-driven SSRNAD (40, 41). In addition, although the direct contribution of IFNγ has not been examined in these models of SSRNAD, IFNγ has been implicated in severe asthma in humans and experimental mouse models (54). Moreover, Th1 responses are well known to suppress Th2 immune responses, which may contribute to the suppression of Th2 responses to AA in this model of SSRNAD (55). Future studies will be required to understand the role of these effector cytokines.

The cellular source of these cytokines remains to be determined but is likely a cooperative effort of adaptive (Th1/Th17) cells and innate cellular sources such as innate lymphoid cells (ILCs). Such innate-like cells are highly plastic in nature, which grants them the ability to alter their functional phenotype in response to various environmental signals (56). For instance, working cooperatively with Th2 cells, ILC2s have emerged as the dominant producer of type 2 cytokines in T2-hi asthma (57, 58). It was previously shown that RAGE was required for rIL-33 or AA induced ILC2 accumulation and type 2 cytokine production in the lungs of mice (29). However, type 1 and 17 cytokines (i.e. IFNγ and IL-17) are elevated in T2-lo asthma (59). While the role of ILC1s in asthma remains largely unknown, ILC3s are elevated in severe asthma and IL-17 producing ILC3s were required for increased airway resistance in a mouse model of obesity-induced asthma (60). A recent report demonstrated that administration of the universal bacterial second messenger, cyclic di-GMP, skewed the response to AA from ILC2s to IFNγ-producing ILC1s (61). Therefore, it is plausible that exposure to AA in the presence of CFA may cause a shift favoring ILC1 and ILC3s in the lung microenvironment along with Th1 and Th17 cells. Such a shift may promote Th1/Th17 immune responses while suppressing the strong Th2 response that is typically elicited by AA. Future investigations are needed to determine how RAGE may influence these intricate innate and adaptive immune responses.

Interestingly, despite the lack of inflammation, humoral immune responses to AA (e.g. development of AA-specific antibodies) remained intact in RAGE^-/-^ mice. This is consistent with a previous study which showed that humoral immunity wasn’t affected by lack of RAGE in mice after chronic HDM exposure (28). In addition, administration of the NLRP3-specific antagonist MCC950 also had no effect on circulating levels of IgE and IgG_1_, indicating that IL-4 driven antibody class switching is independent of RAGE/NLRP3 signaling.

Recent studies have revealed a potential causal role for the NLRP3 inflammasome in severe neutrophilic asthma (15–18). In this study, pharmacological inhibition of NLRP3 significantly reduced allergen-driven neutrophilic responses. Moreover, this study demonstrates that this NLRP3 inflammasome activation and IL-1β elaboration is RAGE-dependent. While some studies have reported roles for RAGE in NLRP3 activation, this is the first study to show RAGE promotes allergen-induced NLRP3 activation and IL-1β release. However, the mechanism by which RAGE promotes NLRP3 inflammasome activation in the AA/CFA model remains unclear.

Mechanistically, DAMP/PRR signaling contributes to both the “priming” and “assembly” steps of NLRP3 inflammasome activation, suggesting RAGE has the potential to be involved in both steps. Specifically, RAGE may be required for the initial induction of NLRP3 inflammasome components or subsequently in the initiation of its assembly. However, this function is not unique to RAGE, as other PRRs with common ligands are also involved (e.g. TLR4). Notably, this study demonstrates that the absence of RAGE diminished NLRP3 inflammasome activation despite the presence of other PRRs involved in the pathway. However, which RAGE-expressing cell-types promote inflammasome activation remains to be determined. While inflammasomes are most abundantly expressed in hematopoietic cells (62, 63), previous studies show that RAGE expression on structural cells promotes the inflammatory response to allergens, potentially through epithelial DAMP release (29, 32). HMGB1 is a prototypical DAMP which binds to RAGE as well as TLR2 and TLR4 and has been linked to inflammasome activation (64, 65). Interestingly, time-course studies revealed AA-induced early biphasic release of HMGB1 into the airways of WT mice, which was nearly undetectable in RAGE^-/-^ mice. It is possible that this early RAGE-dependent HMGB1 release may contribute to the lack of NLRP3 activation in RAGE^-/-^ mice.

Neutrophils recruited to airways in the AA/CFA model exhibited atypical morphology with intense hyper-segmentation as compared to neutrophils recruited by allergen or LPS alone. Hypersegmented neutrophils in BAL of human subjects with obstructive lung disease were associated with increased airflow obstruction (66). *Shin et al.* recently found that diesel exhaust particulate exposure induced recruitment of SiglecF+ neutrophils to the airways of mice, which were more prone to NETosis and exacerbated inflammation compared to SiglecF- neutrophils (51). Moreover Siglec8+ (human homolog to murine SiglecF) were increased in patients with Asthma-COPD overlap. Other studies have reported that SiglecF+ neutrophils have a prolonged lifespan, hypersegmented nuclei, increased mobility and an enhanced inflammatory phenotype (67–71). The current study also found that SiglecF+ neutrophils were present in the airways along with NET accumulation after AA exposure. These SiglecF+ neutrophils were mostly non-viable but persisted in the airways. Studies have shown that incomplete clearance of apoptotic neutrophils causes secondary necrosis, which exacerbates inflammation in the lungs during intense inflammation (72). It is possible that these cells mark mature, late-stage neutrophils, which promote persistent inflammation. NETs produced during sterile and non-sterile inflammation can promote redundant and damaging neutrophilic inflammation (73). These SiglecF+ neutrophils may persist in the airways, eventually producing NETs, further promoting damaging neutrophilic inflammation. RAGE-TLR9 detects extracellular HMGB1-DNA immune-complexes as well as citrullinated peptides in NETs, promoting inflammation (74, 75). Moreover, it was demonstrated that NET-derived HMGB1 induces macrophage pyroptosis and IL-1β release *via* RAGE in a mouse model of sepsis (76). This suggests that RAGE may detect NET-associated components, promoting inflammasome activation and continued neutrophil recruitment. However, it remains to be determined if RAGE influences the process of NETosis or only persistent recruitment.

Time-course experiments revealed that RAGE promoted sustained recruitment of neutrophils into the airways after AA challenge. Neutrophils also have a functional NLRP3 inflammasome and polymorphisms in NLRP3 have been associated with delayed neutrophil apoptosis (77, 78). Therefore, RAGE-dependent inflammasome activation and IL-1β elaboration in early recruited neutrophils may promote persistent neutrophilia seen in WT mice. Future studies are needed to determine if the allergen exposure induces NLRP3 activation in neutrophils and if this is RAGE-dependent. Moreover, it remains to be determined if RAGE directly promotes persistent recruitment or negatively regulates neutrophil clearance.

The role of RAGE in the release of its ligands into the airspaces and the potential contribution to NLRP3 inflammasome activation remains perplexing. The present study has found that RAGE exhibits variable influence on DAMP release. S100A8/A9 is a heterodimeric DAMP, released from cells in response to stress or during cell death, with neutrophils being the primary source (79). Extracellular S100A8/A9 has strong antimicrobial activity and promotes local inflammation through binding of RAGE and TLR4 (80). High levels of S100A8/A9 have been associated with severity of various lung diseases including asthma, COPD and most recently COVID-19 (81–84). Here, the release of S100A8/A9 was independent of RAGE in the AA/CFA model, whereas RAGE was required for S100A8/A9 release when mice were challenged with AA alone. Moreover, we previously found that S100A8/A9 release in response to recombinant IL-5 and IL-13 was also RAGE-dependent (30). This suggests that S100A8/A9 release is promoted by RAGE signaling during Th2-high immune responses but not during Th1/17-high immune responses. S100A8/A9 is a potent stimulator of neutrophil recruitment, which can promote a positive feedback loop of recruiting neutrophils, which in turn release more of the ligand (80). In the AA/CFA model, it is possible that S100A8/A9 promotes persistent neutrophil recruitment through RAGE activation, which is lost in RAGE-null mice, however, S100A8/A9 may also signal through TLR4. Therefore, future studies investigating S100A8/A9 and TLR4 neutralization will be required to determine its potential role in experimental severe asthma. Furthermore, the present study found that early release of HMGB1 into the airways after AA challenge was a RAGE-dependent. A previous study found that RAGE influences the release of HMGB1 from the airway epithelium during allergic sensitization to HDM or cockroach allergen, which involved crosstalk with TLR4 (33). However, these studies examined release at later time-points (24, 48 and 72h post-challenge) during sensitization, whereas the present study examined release prior to 24h after sensitization. AA is known to elicit rapid activation and release of DAMPs such as IL-33 *via* intrinsic protease activity (85). A previous study also found that RAGE promoted HDM or AA induced accumulation of IL-33 in the lungs of mice (29). It is possible that protease activity of AA also promotes rapid release of HMGB1which requires an initial RAGE signal induced through its protease activity. HMGB1 can induce NLRP3 inflammasome components and IL-1β release through collective signaling of TLR2, TLR4 and RAGE (86). However, if early HMGB1 release in response to AA contributes to inflammasome activation and subsequent inflammation remains unclear. It is also possible that other DAMPs, including those associated with NETs (e.g. S100A8/A9 and DNA), promote persistent inflammasome activation upon neutrophil recruitment and induction of NETosis. However, the early initiating factors which promote HMGB1 release and initial inflammasome assembly and activation remain unknown. RAGE binds to a plethora of DAMPs and other molecules (23). Therefore, AA induced damage to the airway epithelium may induce rapid release of other DAMPs such as extracellular DNA or ATP, which can also activate the inflammasome (87). In addition, RAGE activation is well known to induce the rapid production of reactive oxygen species (ROS) which can also stimulate inflammasome activation (88, 89). It also remains unclear if RAGE is required for the priming or activation steps of NLRP3 inflammasome activation. Nonetheless, further investigations are required to elucidate the mechanisms by which RAGE influences DAMP release and how it promotes NLRP3 inflammasome activation in severe asthma.

A potential limitation of the current study is the lack of direct relevance of *M. tuberculosis* (in CFA) to human asthma. However, the mechanisms which drive severe neutrophilic asthma remain poorly understood, making it difficult to model experimentally. Nonetheless, the AA/CFA model of SSRNAD provides a useful experimental tool which induces features similar to that of human neutrophilic asthma. Future studies are warranted to examine the potential role of RAGE in mouse models of severe asthma which better mimic the human scenario by for instance, incorporating allergen exposure with co-existing lung infection or air pollution exposure (15, 90).

Overall, this study further highlights the importance of RAGE in allergen-driven models of asthma and contributes to the understanding of the relationship between RAGE and neutrophilic inflammation. Moreover, this study identified a functional role for RAGE in regulating allergen-induced NLRP3 inflammasome activation in the lungs, which was required for Th1/Th17 cytokine production and airway neutrophilia. This study also demonstrates AA exposure induced recruitment of atypical neutrophils to the airways, which may contribute to sustained severe neutrophilia in the lungs.

## Conclusions

In conclusion, this study reveals a critical role for RAGE in promoting allergen driven SSRNAD in mice through activation of the NLRP3 inflammasome. Taken together with previous studies, this makes RAGE an intriguing potential therapeutic target for not only T2hi but also T2lo asthma.

## Data availability statement

The raw data supporting the conclusions of this article will be made available by the authors, without undue reservation.

## Ethics statement

The animal study was reviewed and approved by University of Pittsburgh’s Institutional Animal Care and Use Committee.

## Author contributions

KK, TO and TP conceptualized the study. KK, JK, ML and TP performed and analyzed experiments. JK, ML and KE provided assistance in execution, analysis and interpretation of flow cytometry. KK and TP wrote the manuscript and KE and TO helped in editing the manuscript. All authors contributed to the article and approved the submitted version.

## Supplementary material

Supplementary Figure S1 (A) WT mice were sensitized to AA extract (100µg) or saline (control) in the presence of CFA on day zero. Mice are then intranasally challenged with saline or AA (25µg) daily on days 14-17 and then euthanized 24h after the final challenge. Mice received an intraperitoneal injection (200µl) of vehicle control (1%DMSO in saline) or Dexamethasone (DEX;2mg/kg) immediately prior to i.n challenge on day 14 and16. (B) Total neutrophils/ml in BALF quantified by total and differential cell counts. (C-H) BALF levels of (C) IL-17A, (D) IFNγ, (E) IL-6, (F) CXCL1, (G) G-CSF and (H) IL-1β. Data are represented as the mean ±SEM, N=10-11 per group. Data are pooled from two independent experiments. *P<0.05 compared to saline + vehicle control group.

Supplementary Figure S2 WT and RAGE^-/-^ were subjected to the AA/CFA model of SSRNAD and BALF examined. Percentages of (A) neutrophils, (B) macrophage/monocytes, (C) eosinophils and (D) lymphocytes. Cell numbers/ml of (E) macrophage/monocytes, (F) lymphocytes and (G) eosinophils. BALF levels of (H) IL-5 and (I) IL-13. N=5-6/group, *P<0.05 for indicated comparison. Data are from a single experiment and are representative of two independent experiments.

Supplementary Figure S3 (A) WT and RAGE^-/-^ mice were sensitized to AA extract (100µg) or saline (control) in the presence of CFA on day zero. Mice are then intranasally challenged with saline or AA (100µg) daily on day 14 and euthanized 24h later. Western blot of whole lung homogenate (B) and BAL cells (C) probed for phospho(p)STAT3, total STAT3 and ACTIN. Each lane represents 3-4 pooled biological replicates from a single experiment. Data are representative of 2 independent experiments.

Supplementary Figure S4 Mice were subjected to the SAL/CFA model. Western blot of whole lung homogenates from WT (left) and RAGE^-/-^ mice (right) for caspase-1 (top), RAGE (middle) and actin (bottom). N=3 (one biological specimen/lane).

Supplementary Figure S5 WT mice were subjected to the AA/CFA model of SSRNAD and were i.n. challenged with saline or AA in the presence of vehicle control or MCC950 (10mg/kg) as depicted in **Figure 4A**. Serum levels of non-specific (A) IgE and (B) IgG_1_ were measured by ELISA. Data are represented as the mean ± SEM. N=5/group. Data are from a single experiment and are representative of two independent experiments. *P<0.05 vs. SAL+VEH.

Supplementary Figure S6 (A) S100A8/S100A9 levels in the BALF of WT and RAGE^-/-^ mice subjected to the AA/CFA model of SSRNAD N=4-8/group data are from a single experiment and are representative of 3 independent experiments. (B) S100A8/S100A9 levels in the BALF of WT and RAGE^-/-^ mice i.n. challenged with saline or AA (25µg) on day 0, 3, 6, 9 and euthanized on day 10. Specimens were from a previously published study, N=8-9/group and are pooled from 2 independent experiments.

Supplementary Figure S7 Representative histograms (% of max) of negative control plots. (A) Fluorescence minus one (FMO) control for anti-SiglecF antibody signal shows no background positivity. (B) Anti-siglecF antibody signal in dead lymphocytes shows no non-specific positivity. (C) Anti-F4/F80 antibody signal shows positive shift for alveolar macrophages (red line) and no non-specific positivity for dead neutrophils (gray). Lymphocytes were gated as live/dead+ FSClow, SSClow and alveolar macrophages were gated as live/dead-, Ly6G-, SiglecF+, CD200R+, CD11c+ and CD206+.
