# Supplementary figures and images for: RAGE contributes to allergen driven severe neutrophilic airway inflammation via NLRP3 inflammasome activation in mice
Source: Front Immunol. 2023 Jan 26;14:1039997. doi: 10.3389/fimmu.2023.1039997 (PMC9910358; doi:10.3389/fimmu.2023.1039997)

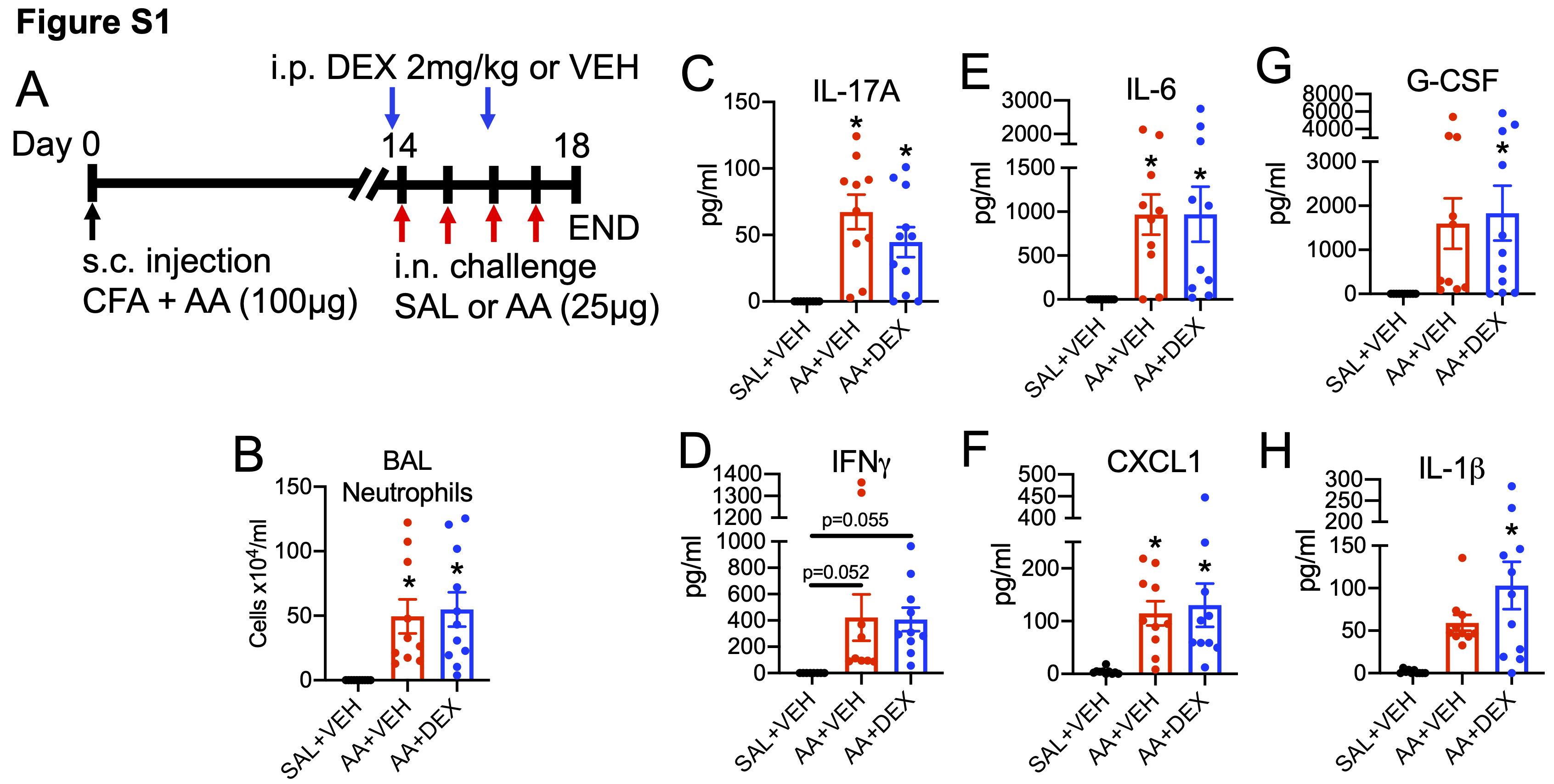

Supplement: Supplementary Figure S1 — (A) WT mice were sensitized to AA extract (100µg) or saline (control) in the presence of CFA on day zero. Mice are then intranasally challenged with saline or AA (25µg) daily on days 14-17 and then euthanized 24h after the final challenge. Mice received an intraperitoneal injection (200µl) of vehicle control (1%DMSO in saline) or Dexamethasone (DEX;2mg/kg) immediately prior to i.n challenge on day 14 and16. (B) Total neutrophils/ml in BALF quantified by total and differential cell counts. (C-H) BALF levels of (C) IL-17A, (D) IFNγ, (E) IL-6, (F) CXCL1, (G) G-CSF and (H) IL-1β. Data are represented as the mean ±SEM, N=10-11 per group. Data are pooled from two independent experiments. *P<0.05 compared to saline + vehicle control group. [file Image_1.jpeg]

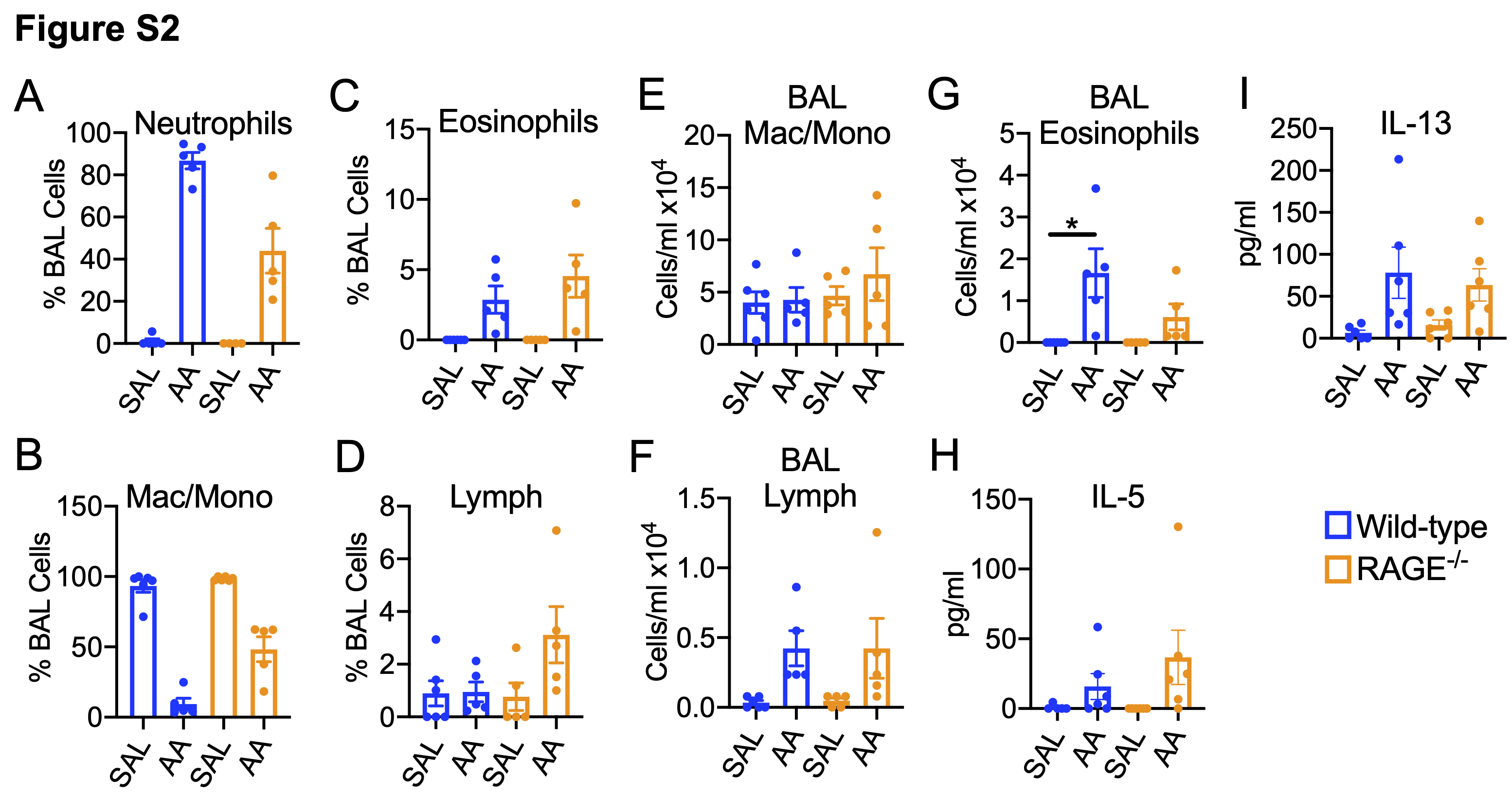

Supplement: Supplementary Figure S2 — WT and RAGE-/- were subjected to the AA/CFA model of SSRNAD and BALF examined. Percentages of (A) neutrophils, (B) macrophage/monocytes, (C) eosinophils and (D) lymphocytes. Cell numbers/ml of (E) macrophage/monocytes, (F) lymphocytes and (G) eosinophils. BALF levels of (H) IL-5 and (I) IL-13. N=5-6/group, *P<0.05 for indicated comparison. Data are from a single experiment and are representative of two independent experiments. [file Image_2.jpeg]

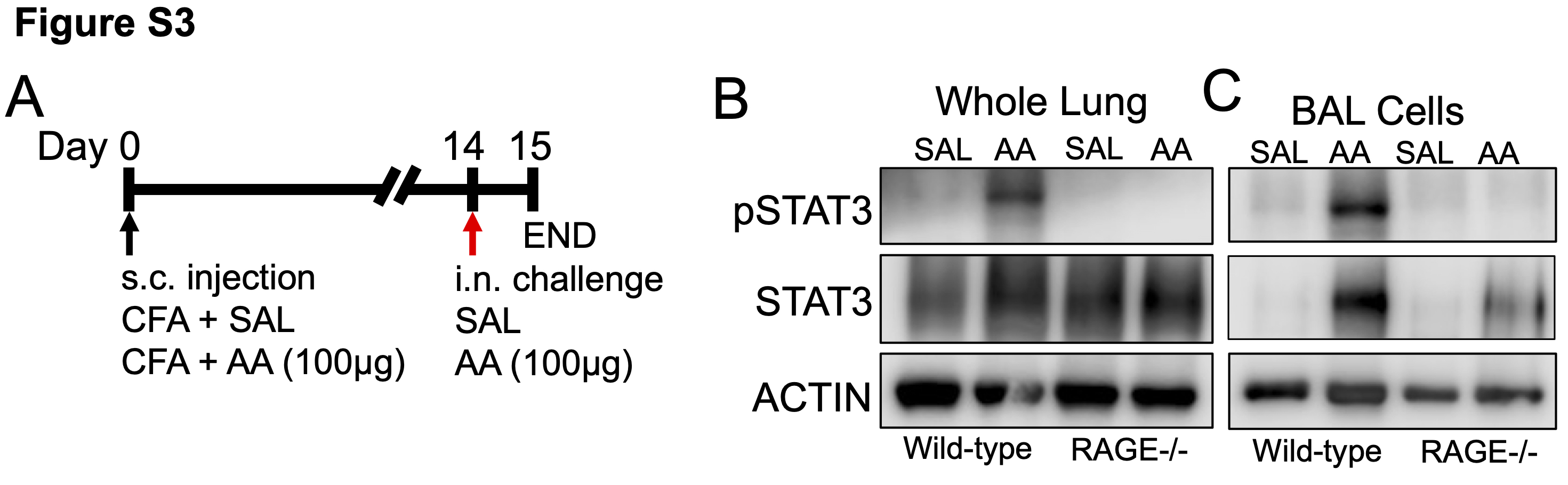

Supplement: Supplementary Figure S3 — (A) WT and RAGE-/- mice were sensitized to AA extract (100µg) or saline (control) in the presence of CFA on day zero. Mice are then intranasally challenged with saline or AA (100µg) daily on day 14 and euthanized 24h later. Western blot of whole lung homogenate (B) and BAL cells (C) probed for phospho(p)STAT3, total STAT3 and ACTIN. Each lane represents 3-4 pooled biological replicates from a single experiment. Data are representative of 2 independent experiments. [file Image_3.jpeg]

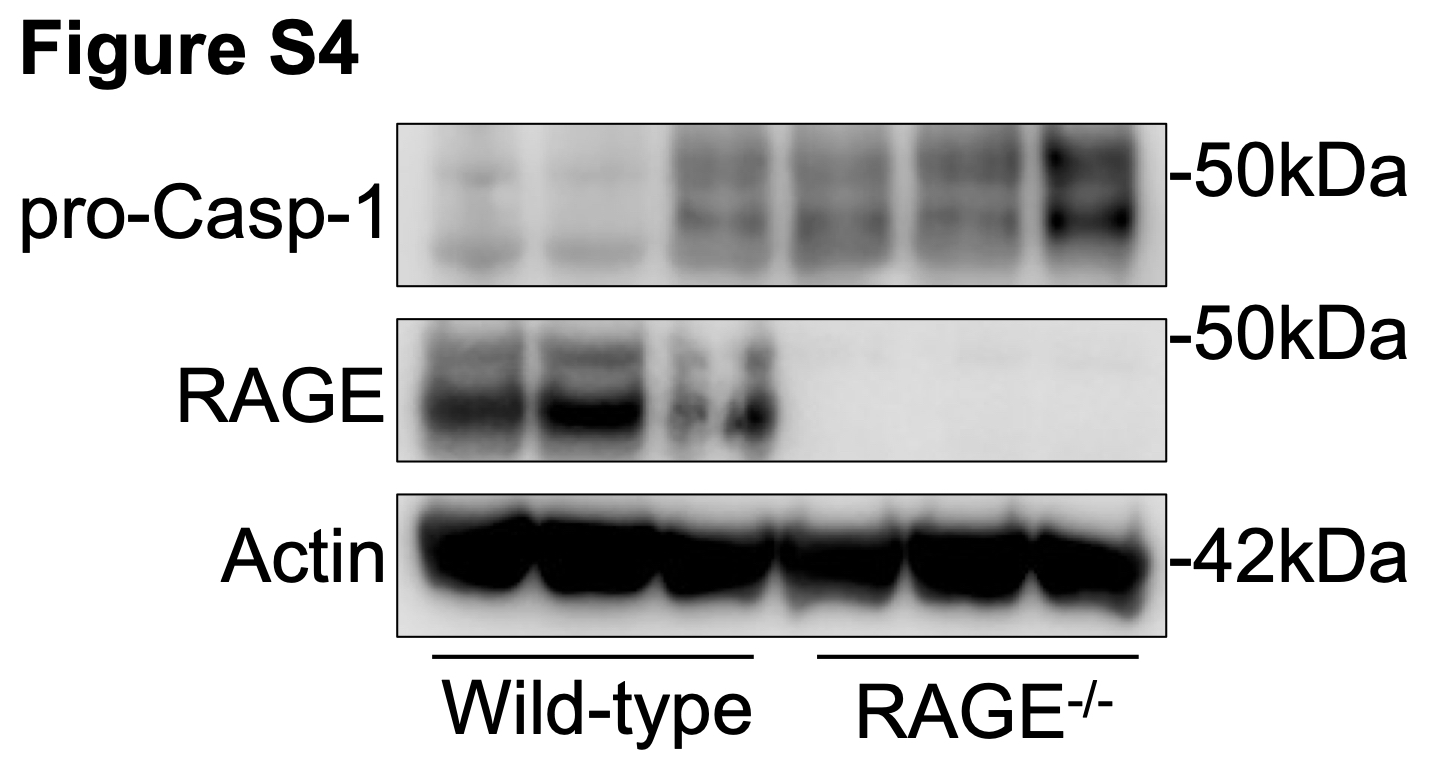

Supplement: Supplementary Figure S4 — Mice were subjected to the SAL/CFA model. Western blot of whole lung homogenates from WT (left) and RAGE-/- mice (right) for caspase-1 (top), RAGE (middle) and actin (bottom). N=3 (one biological specimen/lane). [file Image_4.jpeg]

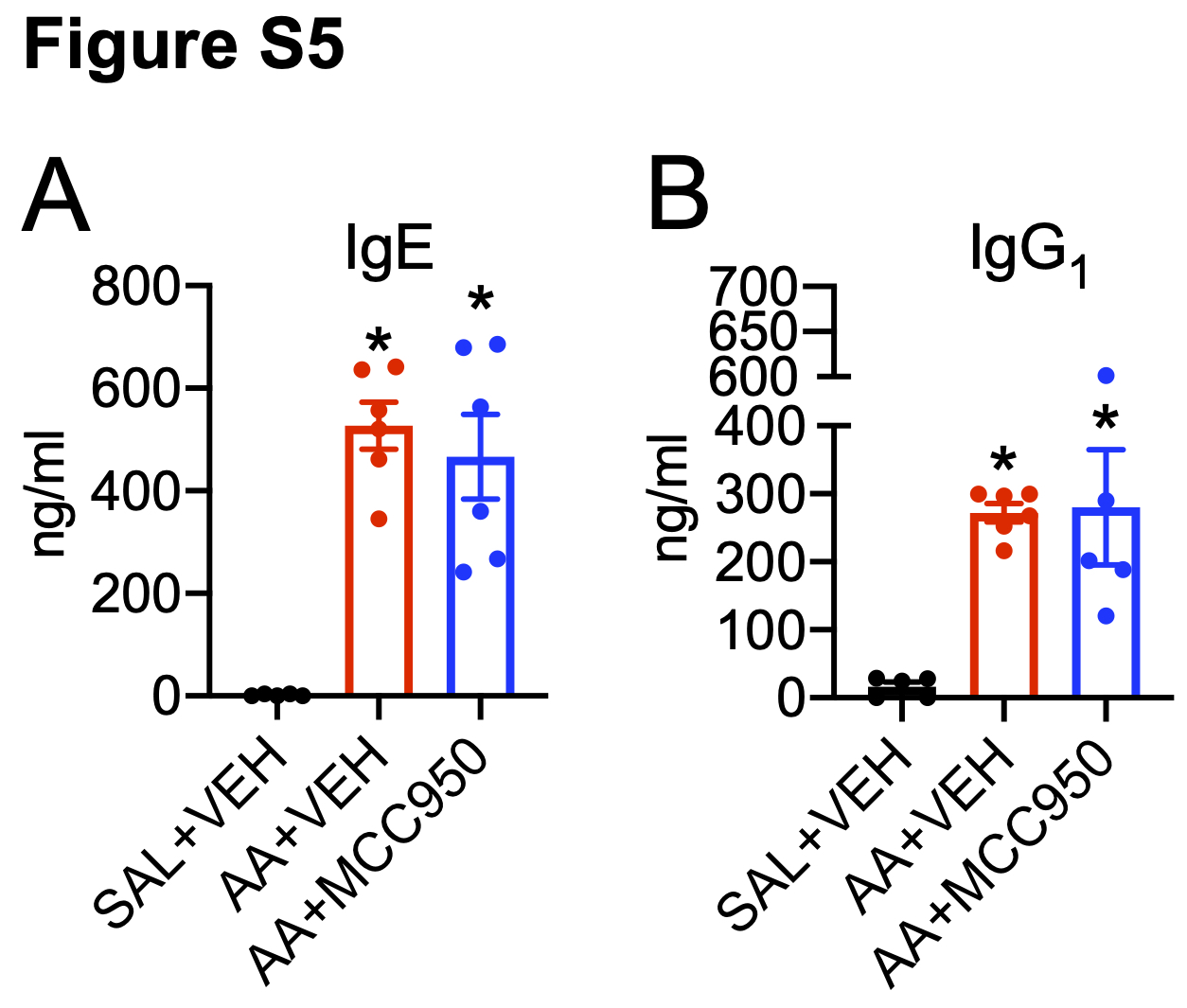

Supplement: Supplementary Figure S5 — WT mice were subjected to the AA/CFA model of SSRNAD and were i.n. challenged with saline or AA in the presence of vehicle control or MCC950 (10mg/kg) as depicted in Figure 4A . Serum levels of non-specific (A) IgE and (B) IgG1 were measured by ELISA. Data are represented as the mean ± SEM. N=5/group. Data are from a single experiment and are representative of two independent experiments. *P<0.05 vs. SAL+VEH. [file Image_5.jpeg]

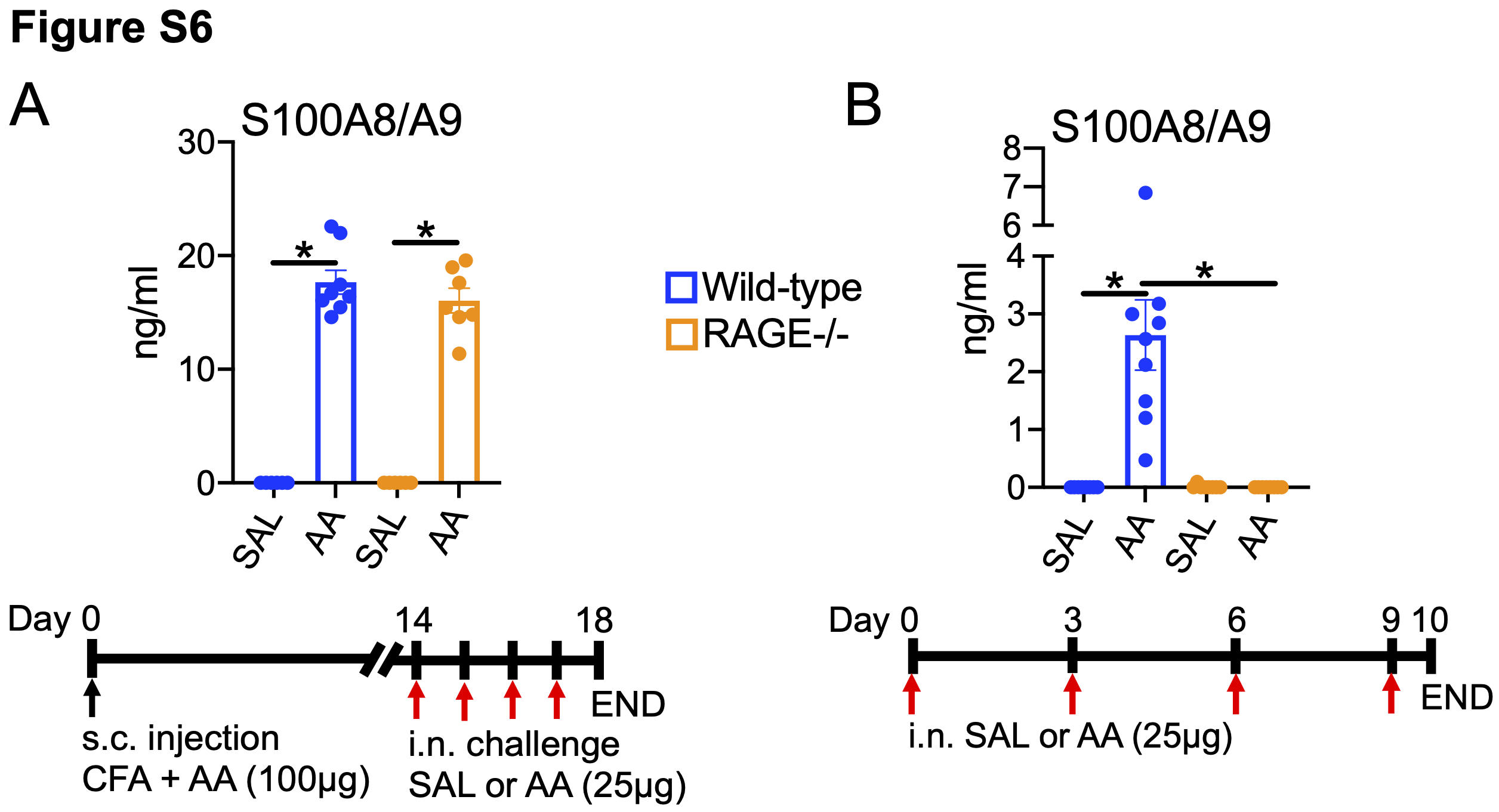

Supplement: Supplementary Figure S6 — (A) S100A8/S100A9 levels in the BALF of WT and RAGE-/- mice subjected to the AA/CFA model of SSRNAD N=4-8/group data are from a single experiment and are representative of 3 independent experiments. (B) S100A8/S100A9 levels in the BALF of WT and RAGE-/- mice i.n. challenged with saline or AA (25µg) on day 0, 3, 6, 9 and euthanized on day 10. Specimens were from a previously published study, N=8-9/group and are pooled from 2 independent experiments. [file Image_6.jpeg]

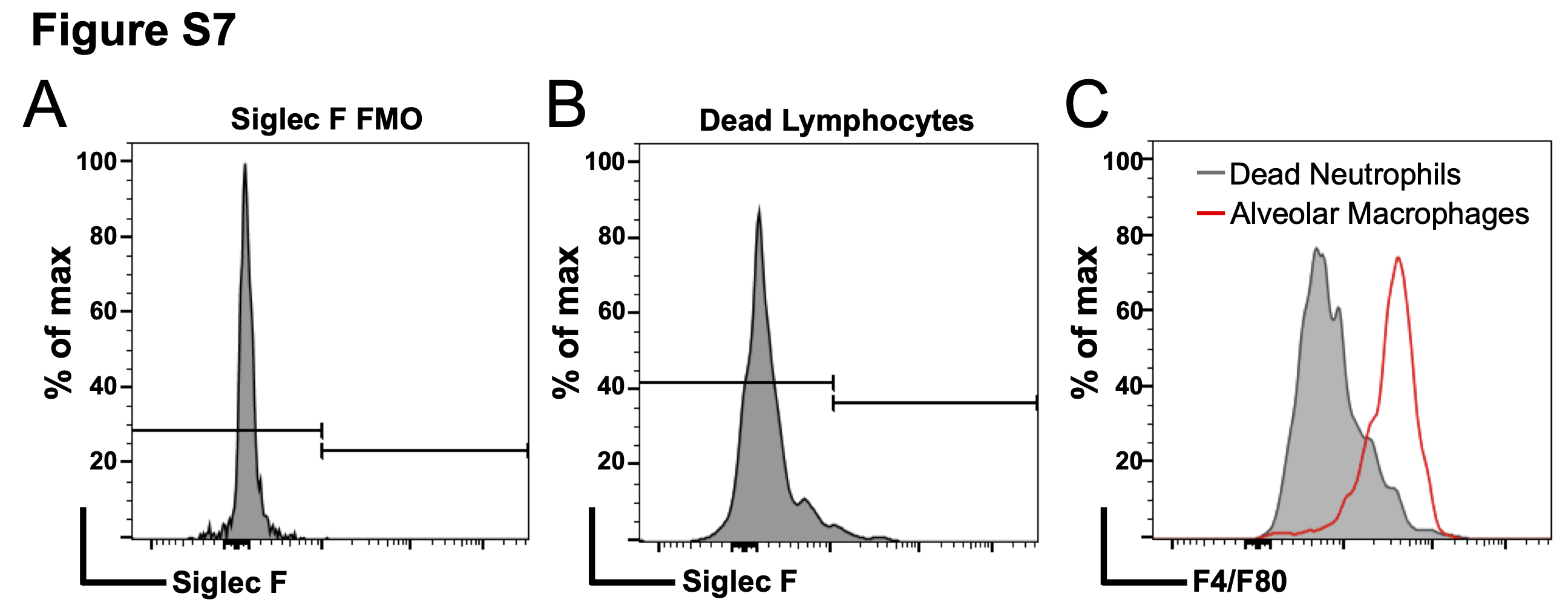

Supplement: Supplementary Figure S7 — Representative histograms (% of max) of negative control plots. (A) Fluorescence minus one (FMO) control for anti-SiglecF antibody signal shows no background positivity. (B) Anti-siglecF antibody signal in dead lymphocytes shows no non-specific positivity. (C) Anti-F4/F80 antibody signal shows positive shift for alveolar macrophages (red line) and no non-specific positivity for dead neutrophils (gray). Lymphocytes were gated as live/dead+ FSClow, SSClow and alveolar macrophages were gated as live/dead-, Ly6G-, SiglecF+, CD200R+, CD11c+ and CD206+. [file Image_7.jpeg]
